# Possible beneficial association between renin-angiotensin-aldosterone-system blockade usage and graft prognosis in allograft IgA nephropathy: a retrospective cohort study

**DOI:** 10.1186/s12882-019-1537-1

**Published:** 2019-09-11

**Authors:** Sehoon Park, Chung Hee Baek, Heounjeong Go, Young Hoon Kim, Sang–il Min, Jongwon Ha, Yong Chul Kim, Jung Pyo Lee, Yon Su Kim, Kyung Chul Moon, Su-Kil Park, Hajeong Lee

**Affiliations:** 1 Department of Internal Medicine, Seoul National University Hospital, Seoul, South Korea; 2 Department of Biomedical Sciences, Seoul National University College of Medicine, Seoul, South Korea; 3 Department of Internal Medicine, Asan Medical Center, University of Ulsan College of Medicine, Seoul, South Korea; 4 Department of Pathology, Asan Medical Center, University of Ulsan College of Medicine, Seoul, South Korea; 5 Department of Surgery, Asan Medical Center, University of Ulsan College of Medicine, Seoul, South Korea; 6 Department of Surgery, Seoul National University Hospital, Seoul National University College of Medicine, Seoul, South Korea; 7 Kidney Research Institute, Seoul National University College of Medicine, Seoul, South Korea; 8 Department of Internal Medicine, Seoul National University Boramae Medical Center, Seoul, South Korea; 9 Department of Pathology, Seoul National University Hospital, Seoul, South Korea

**Keywords:** Transplantation, Allograft, IgA nephropathy, Renin-angiotensin-aldosterone system, ACE inhibitors, Angiotensin receptor blockade, Proteinuria

## Abstract

**Background:**

Although immunoglobulin A nephropathy (IgAN) is associated with an increased risk of renal allograft failure, evidences for its treatment, including renin-angiotensin-aldosterone system blockade (RAASB) usage, remain limited.

**Methods:**

In this bi-center retrospective cohort study, we included patients who were recently diagnosed with IgAN through allograft biopsies. We identified their 6-month antihypertensive medication prescriptions and investigated the association between the medication types, albuminuria changes, and risk of 5-year death-censored-graft-failure (DCGF). The mixed effect model and cox regression analysis were used.

**Results:**

A total of 464 allograft IgAN patients were included: 272, 38, 33, and 121 patients in the no antihypertensive medication, single agent RAASB, single agent beta blocker (BB)/calcium channel blocker (CCB), and combination therapy groups, respectively. High-degree albuminuria after 6 months of allograft IgAN diagnosis was an important prognostic parameter and a partial mediator for the association between the subgroups and 5-year DCGF. The usage of single RAASB was associated with decrement of albuminuria from allograft IgAN diagnosis (*P* for interaction = 0.03). The single BB/CCB group demonstrated significantly worse prognosis than the single RAASB group (adjusted hazard ratio, 2.76 [1.09–6.98]; *P* = 0.03).

**Conclusions:**

In conclusion, RAASB may be beneficial for graft prognosis in early allograft IgAN patients who require single antihypertensive medication therapy, by means of reducing albuminuria. Further investigation of treatment strategy in allograft IgAN is warranted.

**Electronic supplementary material:**

The online version of this article (10.1186/s12882-019-1537-1) contains supplementary material, which is available to authorized users.

## Background

Immunoglobulin A nephropathy (IgAN), the most common primary glomerulonephritis during both the pre- and post-transplant eras [1–3], has demonstrated better post-transplant prognosis than other etiologies of end-stage renal disease (ESRD) in the short-term [4]. However, the development or recurrence of IgAN in allograft is associated with an increased risk of graft failure in the long-term [5, 6]. Although this accelerated graft dysfunction has been repetitively observed [4, 7, 8], evidence for treatment strategies of allograft IgAN remains limited. As allograft glomerulonephritis is one of the major obstacles in the improvement of long-term graft outcome in modern transplant medicine, the development of an adequate management strategy for allograft IgAN would be beneficial for prolonged graft use.

Renin-angiotensin-aldosterone system blockade (RAASB), which includes angiotensin converting enzyme inhibitors and angiotensin receptor blockades, has been shown to effectively suppress the progression of native IgAN [9–12]. However, the benefits of RAASB during post-transplant periods are still debatable [13–16], although this is mainly related to a low prescription rate of the medications in the post-transplantation period [15]. In addition, whether RAASB usage is associated with better prognosis for primary glomerulonephritis in allograft, including IgAN [17, 18], is uncertain, even though a clinical guideline recommend its usage with a low-grade recommendation level [19].

In this study, we aimed to provide supporting evidence for the possible benefits of RAASB in allograft IgAN. We reviewed our retrospective cohort of allograft IgAN patients and identified their prescribed hypertensive medication categories [8, 20]. We then compared their prognosis in terms of death-censored-graft-failure (DCGF) and changes in albuminuria levels.

## Methods

### Study design and study population

This was a retrospective cohort study performed in two tertiary referral hospitals in Korea. We included transplant recipients who were diagnosed with pathologically confirmed allograft IgAN. The exclusion criteria were as follows: 1) patients who were diagnosed with allograft IgAN before the year 2000, as electronic medical records did not provide sufficient information related to patients’ prescribed medications during this period, and 2) patients with follow-up loss or acute graft failure within 6 months from allograft IgAN diagnosis. The remaining patients were stratified into 4 study groups: 1) those who did not receive antihypertensive medications (no medication group); 2) those who received single RAASB-category medications (single RAASB group); 3) those who received single beta-blocker (BB) or calcium channel blocker (CCB) (single BB/CCB group); and 4) those who received multiple categories of medications (combination group). In this studied cohort, no patient received other single agent therapy apart from RAASB, CCB, or BB. A medication history was considered present when an according category was prescribed as a maintenance medication for targeting blood pressure (BP) control within 6 months of allograft IgAN diagnosis.

### Allograft IgAN and graft biopsy

Biopsies to confirm allograft IgAN were mostly performed under the following clinical criteria: a progressive decline in renal function, persistent hematuria, or significant proteinuria of more than 1.0 g/day [7, 8]. Some patients underwent protocol biopsies at time-zero and then again 2 weeks post-transplantation. The pathologic diagnosis of allograft IgAN was confirmed when the specimens demonstrated typical immunofluorescence pattern of IgA ± C3 staining in the glomerulus mesangial space.

### Data collection

Patients’ clinical characteristics at the time of diagnosis for allograft IgAN were collected, including age, sex, and the relationship between the donor and recipient. Laboratory values, including estimated glomerular filtration rate (eGFR) using the CKD-EPI equation, dipstick albuminuria (none or trace, 1+, or ≥ 2+), [21] and mean arterial pressure (MAP, 1/3 of systolic BP plus 2/3 of diastolic BP) were collected at the time of biopsy and after 6 months. As RAASB tends to be prescribed to those without progressive decline in graft function in view of its association with acute kidney injury, we calculated the time-averaged eGFRs during the first 3-month-period from allograft IgAN diagnosis, in order to include this variable in our multivariable analysis. The duration from transplantation to allograft IgAN diagnosis was collected, considering that allograft IgAN is a strong time-dependent event [8]. Information on coexisting evidence of acute rejection was also recorded from the pathology reports. Specialized pathologists additionally reviewed the updated Oxford classification, which showed clinical significance also in allograft IgAN [20], in the pathology slides including; M (mesangial hypercellularity), E (endocapillary hypercellularity), S (segmental sclerosis), T (interstitial fibrosis or tubular atrophy), and C (cellular or fibrocellular crescent formation). There were missing cases regarding the Oxford classification because some slides were missing and some were at insufficient quality for additional pathology review.

### Study outcome

The main study outcome was 5-year DCGF. The follow-up period was the duration from allograft IgAN diagnosis until DCGF outcome, follow-up loss, or 5 years. DCGF was defined as a return to maintenance dialysis or re-transplantation, and death events were censored.

### Statistical analysis

We presented categorical variables as frequencies (percentages) and continuous variables as median values (interquartile ranges). The Kruskal-Wallis test was used to compare continuous variables between the study groups, and the chi-squared tests for the categorical variables. We plotted Kaplan-Meier survival curves to demonstrate survival data, and the log-rank test was used to calculate the *p*-values. The mixed effect model was used to investigate whether usage of a single RAASB had significant interaction with changes in albuminuria or MAP at allograft IgAN diagnosis and after 6 months among those who received antihypertensive medication. We used univariable and multivariable Cox regression analysis to analyze DCGF outcome, and the following variables were included in the multivariable models, if not otherwise specified: age, sex, CKD-EPI eGFR (categorical, < 15, ≥15 and 30, ≥30 and < 45, ≥45 and < 60, ≥60), albuminuria (none or trace, 1+, and ≥ 2+), mean arterial pressure (mmHg), and presence of acute rejection at the time of recurrence. In addition, considering that a difference in longitudinal clinical course might have been present among the studied groups, additional multivariable model analysis was performed, changing the eGFR value at baseline to the time-averaged value within 3 months from allograft IgAN diagnosis. In the multivariable analysis testing the association between the high-degree albuminuria after 6 months and the risk of DCGF in patients without prescribed antihypertensive medications, the eGFR and MAP at 6 months were included in the multivariable model, instead of values at baseline. Whether the presence of high-degree albuminuria was a partial mediator with significance for the association between the antihypertensive medication subgroups and 5-year DCGF was tested using the “mediation” package [22] in R (version 3.4.2, The R Foundation). For the missing values, as they appeared in a random manner, we performed multivariate imputation using classification and the regression trees method with the “mice” package in R [23]. All other statistical analyses were also performed with R. A two-sided *p* value of less than 0.05 was considered to indicate statistical significance.

## Results

### Study population

**Fig. 1:**
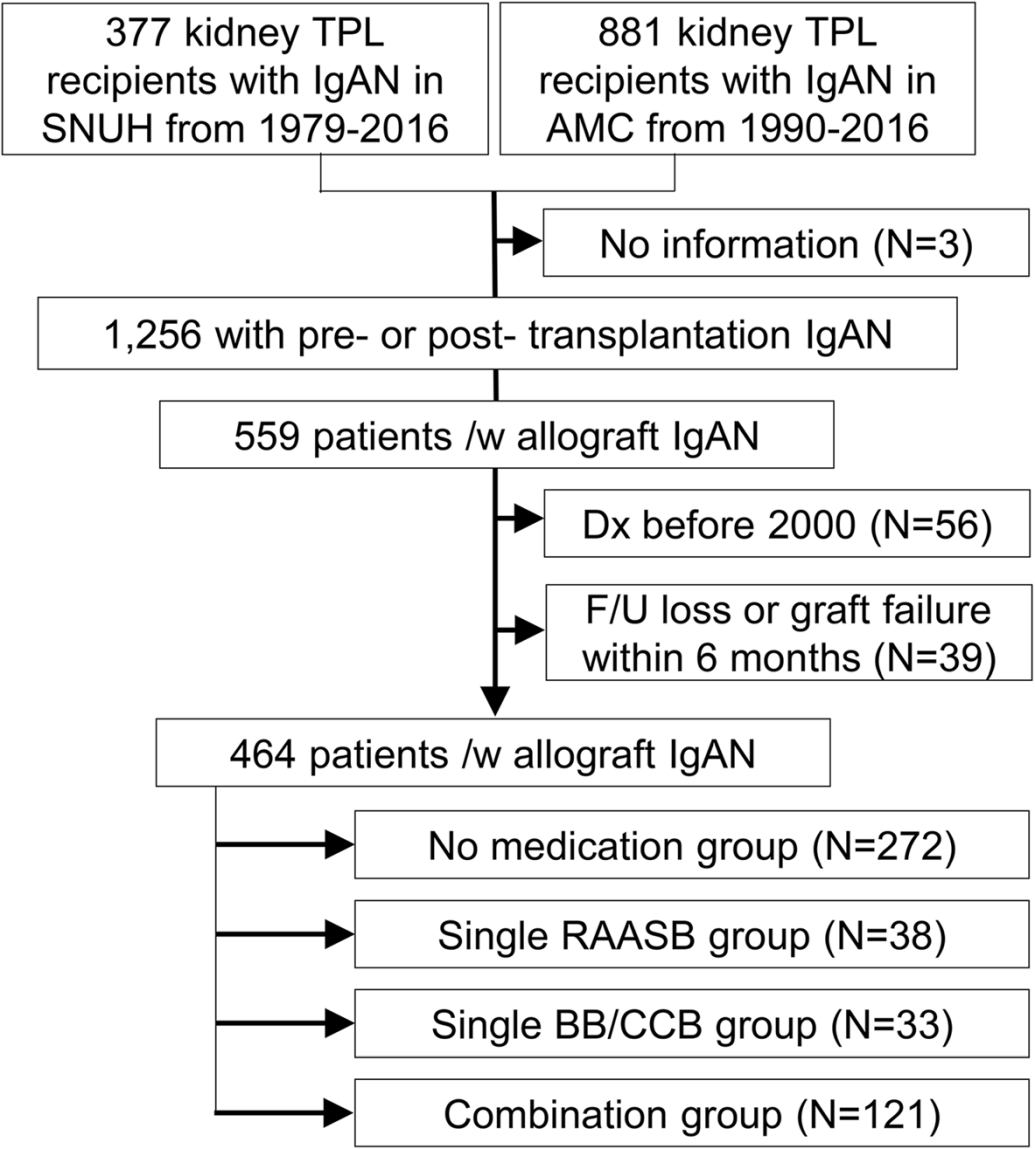
Study population

Of the 559 allograft IgAN patients, 464 patients were included in the current study after exclusion criteria were applied (Fig. 1). Among them, 100 patients were determined to have initial native IgAN as the cause of ESRD, and 11 had other kidney disease, suggesting possible de novo cases. The other 353 patients had unknown primary etiology or only clinical diagnoses for ESRD. Regarding antihypertensive medication usage, there were 272, 38, 33, and 121 allograft IgAN patients in the no medication, single RAASB, single BB/CCB, and combination groups, respectively.

### Characteristics of the study population

Significant differences in baseline characteristics were found between the study groups (Table 1). Although the age and sex distributions were similar between the groups, the single RAASB group had a significantly longer duration from transplant to allograft IgAN diagnosis. This group had favorable clinical characteristics regarding higher eGFR values and less frequent coexisting acute rejection when they were diagnosed. On the other hand, a substantially higher portion of patients in the single RAASB group had albuminuria and hematuria at baseline. Other medication usage, including tacrolimus prescription ratio among the used calcineurin inhibitors, steroid, or mycophenolic acid, did not differ largely between the studied groups.

**Table 1:** Comparisons of baseline characteristics at the time of allograft IgAN diagnosis according to anti-hypertensive medication prescription

	No medication	Single RAASB	Single BB/CCB	Combination
	(*N*=272)	(*N*=38)	(*N*=33)	(*N*=121)
Age at allograft IgAN Dx (years)	44 [34-50]	41 [36-47]	44 [32-54]	43 [35-51]
Male Sex	181 (66.5%)	22 (57.9%)	25 (75.8%)	83 (68.6%)
Duration from transplant to allograft IgAN diagnosis (years)	3.8 [ 0.7- 7.5]	6.7 [ 3.2- 9.2]	5.4 [ 2.8- 8.1]	5.1 [ 2.0- 9.3]
Blood pressure (mmHg)				
Systolic BP	129.0 [117.0-138.0]	120.0 [110.0-133.0]	120.0 [115.0-136.0]	129.0 [118.5-139.0]
Diastolic BP	78.0 [70.0-86.0]	74.0 [65.0-80.0]	78.0 [70.0-85.0]	80.0 [70.0-88.0]
MAP	94.3 [85.3-101.3]	84.8 [79.7-92.7]	93.0 [85.7-100.7]	94.3 [85.0-101.0]
Laboratory values				
Serum creatinine (mg/dL)	1.7 [ 1.4- 2.0]	1.5 [ 1.2- 2.1]	1.9 [ 1.6- 2.6]	1.7 [ 1.4- 2.2]
eGFR (mL/min/1.73 m^2^)	45.0 [35.4-57.0]	50.9 [36.9-70.3]	38.0 [27.3-45.9]	45.1 [33.8-60.5]
Albuminuria				
None or trace	189 (69.7%)	9 (23.7%)	20 (60.6%)	42 (35.0%)
1+	27 (10.0%)	8 (21.1%)	7 (21.2%)	17 (14.2%)
=2+	55 (20.3%)	21 (55.3%)	6 (18.2%)	61 (50.8%)
Hematuria	18 (6.6%)	18 (47.4%)	6 (18.2%)	41 (33.9%)
Other medication usage				
Tacrolimus	138 (50.7)	16 (42.1)	20 (60.6)	51 (42.1)
Steroid	257 (94.5)	35 (92.1)	31 (93.9)	112 (92.6)
Mycophenolic acid	182 (66.9)	30 (78.9)	24 (72.7)	94 (77.7)
Donor characteristics				
Age	39 [31-47]	36 [30-42]	40 [28-47]	40 [30-48]
Male sex	151 (55.5%)	21 (55.3%)	16 (48.5%)	61 (50.8%)
Type of donor				
Living related	157 (57.7%)	24 (63.2%)	16 (48.5%)	72 (59.5%)
Living unrelated	60 (22.1%)	9 (23.7%)	12 (36.4%)	23 (19.0%)
Deceased	55 (20.2%)	5 (13.2%)	5 (15.2%)	26 (21.5%)
Pathological findings				
Coexisting acute rejection	94 (34.6%)	5 (13.2%)	11 (33.3%)	33 (27.3%)
Oxford classification				
Mesangial hypercellularity				
M0	142 (81.6%)	23 (79.3%)	18 (81.8%)	56 (67.5%)
M1	32 (18.4%)	6 (20.7%)	4 (18.2%)	27 (32.5%)
Endocapillary hypercellularity				
E0	130 (78.3%)	16 (57.1%)	16 (72.7%)	49 (59.8%)
E1	36 (21.7%)	12 (42.9%)	6 (27.3%)	33 (40.2%)
Segmental sclerosis				
S0	108 (61.7%)	14 (48.3%)	16 (72.7%)	43 (51.2%)
S1	67 (38.3%)	15 (51.7%)	6 (27.3%)	41 (48.8%)
Tubular atrophy/interstitial fibrosis				
T0	127 (72.6%)	21 (72.4%)	14 (63.6%)	56 (67.5%)
T1	33 (18.9%)	4 (13.8%)	4 (18.2%)	17 (20.5%)
T2	15 (8.6%)	4 (13.8%)	4 (18.2%)	10 (12.0%)
Cellular/fibrocellular crescents				
C0	239 (87.9%)	30 (78.9%)	29 (87.9%)	88 (72.7%)
C1	30 (11.0%)	7 (18.4%)	4 (12.1%)	29 (24.0%)
C2	3 (1.1%)	1 (2.6%)	0 (0.0%)	4 (3.3%)

*RAASB* Renin-angiotensin-aldosterone system blockades, *BB* Beta blockers, *CCB* Calcium channel blockers, *IgAN* Immunoglobulin A nephropathy, *eGFR* Estimated glomerular filtration rate, *BP* Blood pressure, *MAP* Mean arterial pressure

### Risk factors for 5-year DCGF in the study population

The risk factors for 5-year DCGF in the study population are shown in Table 2. Male sex, presence of T in the Oxford classification, and impairment of kidney function, as reflected by decrease in eGFR, were factors prominently associated with an increased risk of DCGF. Particularly, high-degree albuminuria after 6 months of allograft IgAN diagnosis was associated with very high risk for DCGF, which were even comparable to the categorical eGFR variable of < 30 or < 15 mL/min/1.73 m^2^.

**Table 2:** Risk factors for DCGF in the study cohort

	^a^Adjusted HR (95% CI)	*P*
Age (years)	1.00 (0.97–1.02)	0.71
Male sex (.vs female)	2.50 (1.46–4.28)	< 0.001
Duration from transplant to allograft IgAN diagnosis (years)	1.05 (0.98–1.12)	0.18
eGFR (.vs ≥ 60 mL/min/1.73 m^2^)		
≥ 45 and < 60	1.61 (0.68–3.82)	0.28
≥ 30 and < 45	2.35 (1.02–5.38)	0.04
≥ 15 and < 30	6.88 (2.94–16.14)	< 0.001
< 15	7.43 (2.28–24.17)	< 0.001
Albuminuria (.vs none or trace)		
1+ at baseline	0.95 (0.43–2.10)	0.90
≥ 2+ at baseline	1.56 (0.86–2.83)	0.15
1+ at 6 months	1.81 (0.82–3.98)	0.14
≥ 2+ at 6 months	5.64 (3.20–9.93)	< 0.001
MAP (mmHg)		
High MAP (> 100 mmHg) at baseline	1.12 (0.67–1.90)	0.66
High MAP (> 100 mmHg) at 6 months	1.13 (0.72–1.77)	0.59
Coexisting acute rejection	1.07 (0.65–1.76)	0.80
Oxford classification		
M1 (vs. M0)	1.09 (0.59–2.03)	0.78
E1 (vs. E0)	1.62 (0.89–2.98)	0.11
S1 (vs. S0)	1.36 (0.49–3.80)	0.52
T1 or T2 (vs. T0)	2.08 (1.21–3.55)	0.008
C1 or C2 (vs. C0)	1.50 (0.88–2.54)	0.13

*DCGF* Death-censored-graft failure, *IgAN* Immunoglobulin A nephropathy, *eGFR* Estimated glomerular filtration rate, *MAP* Mean arterial pressure

^a^Adjusted with all variables in the table. When albuminuria or MAP after 6 months were included in the model, the values of eGFR, MAP, and the degree of albuminuria at 6 months from diagnosis were included instead the baseline values. Missing values were imputed using the multivariate imputation using classification and the regression trees method

Among those who did not require antihypertensive medications, the degree of albuminuria at baseline was not significantly associated with the risk of 5-year DCGF (Additional file 1: Figure S1). However, those with persistent albuminuria or who developed high-degree (≥2+) albuminuria after 6 months demonstrated an increased risk of 5-year DCGF. This association was significant even after adjustment for age, sex, time from transplantation to allograft IgAN diagnosis, and eGFR and MAP values after 6 months (adjusted HR, 6.70 [1.51–29.76]; *P* = 0.01). Meanwhile, the presence of high MAP was not significantly associated with 5-year DCGF in this patient subgroup, suggesting the relative importance of the high-degree albuminuria after 6 months.

By contrast, those who received antihypertensive medications following the diagnosis of allograft IgAN exhibited worse prognosis than those who did not (Additional file 2: Figure S2). Albuminuria and presence of high MAP demonstrated a significant association with 5-year DCGF risk in this patient group; however, baseline high-degree albuminuria lost its significance in multivariable analysis (adjusted HR, 1.31 [0.77–2.23]; *P* = 0.32). High-degree albuminuria at 6 months remained a significant predictive factor associated with 5-year DCGF in this subgroup (adjusted HR, 2.77 [1.56–4.90]; *P* <  0.001), also showing the persistent high-degree albuminuria was an important risk factor for DCGF.

### Blood pressure and urine dipstick albuminuria according to antihypertensive medications

We analyzed whether differences were present in the urine dipstick results of those with available values both at the time of allograft IgAN diagnosis and after 6 months (Table 3). Compared to the other groups, the single RAASB group had a lower portion of patients with persistent albuminuria or who had developed high-degree albuminuria after 6 months. By contrast, the single BB/CCB group had a higher portion of allograft IgAN patients who developed high-degree (≥2+) dipstick albuminuria at 6 months. Moreover, these differences in changes of albuminuria presence were significant (P for interaction = 0.03) with single RAASB usage even among those who received single antihypertensive medications, showing that RAASB may be better regarding albuminuria reduction than other drug types.

**Table 3:** Albuminuria and blood pressures at the time of diagnosis and after 6 months of allograft IgAN patients who had complete information

	At allograft IgAN diagnosis			After 6 months		
Dipstick albuminuria	None or trace	1+	≥2+	None or trace	1+	≥2+
No medication (*N* = 216)	143 (66.2%)	22 (10.2%)	51 (23.6%)	166 (76.9%)	26 (12.0%)	24 (11.1%)
Single RAASB (N = 38)	9 (23.7%)	8 (21.1%)	21 (55.3%)	18 (47.4%)	7 (18.4%)	13 (34.2%)
Single BB/CCB (*N* = 28)	19 (67.9%)	5 (17.9%)	4 (14.3%)	15 (53.6%)	4 (14.3%)	9 (32.1%)
Combination (*N* = 94)	34 (36.2%)	10 (10.6%)	50 (53.2%)	33 (35.1%)	18 (19.1%)	43 (45.7%)
Blood pressure	Systolic BP	Diastolic BP	MAP	Systolic BP	Diastolic BP	MAP
No medication (*N* = 268)	129.0 [117.0–138.0]	78.0 [70.0–85.5]	95.0 [85.5–102.7]	125.0 [116.0–135.0]	76.0 [68.0–84.0]	92.7 [84.5–100.2]
Single RAASB (N = 33)	120.0 [110.0–133.0]	74.0 [65.0–80.0]	88.7 [81.7–98.7]	122.0 [118.0–136.0]	81.0 [72.0–88.0]	94.7 [90.0–103.3]
Single BB/CCB (N = 28)	120.0 [114.5–136.0]	79.0 [69.5–87.0]	93.2 [84.5–100.3]	126.5 [116.5–140.0]	79.0 [70.5–84.0]	94.2 [85.8–103.3]
Combination (*N* = 104)	129.0 [118.5–139.5]	80.0 [70.0–88.0]	96.3 [88.0–103.3]	128.0 [116.0–140.0]	79.0 [70.0–87.0]	93.7 [85.0–103.8]

*IgAN* Immunoglobulin A nephropathy, *RAASB* Renin-angiotensin-aldosterone blockades, *BB* Beta blockers, *CCB* Calcium channel blockers, *BP* Blood pressure, *MAP* Mean arterial pressure

In terms of blood pressure values, the median MAP was the lowest in the single RAASB group; however, the difference was not statistically significant (*P* = 0.09). In fact, the MAP values became even more similar between the studied groups after 6 months. The changes in high MAP (> 100 mmHg) presence (P for interaction = 0.11) or MAP values (P for interaction = 0.28) did not seem to be affected by use of single RAASB. Therefore, we could not identify a significant difference in blood pressure control according to the antihypertensive medication types.

### DCGF according to antihypertensive medication categories

**Fig. 2:**
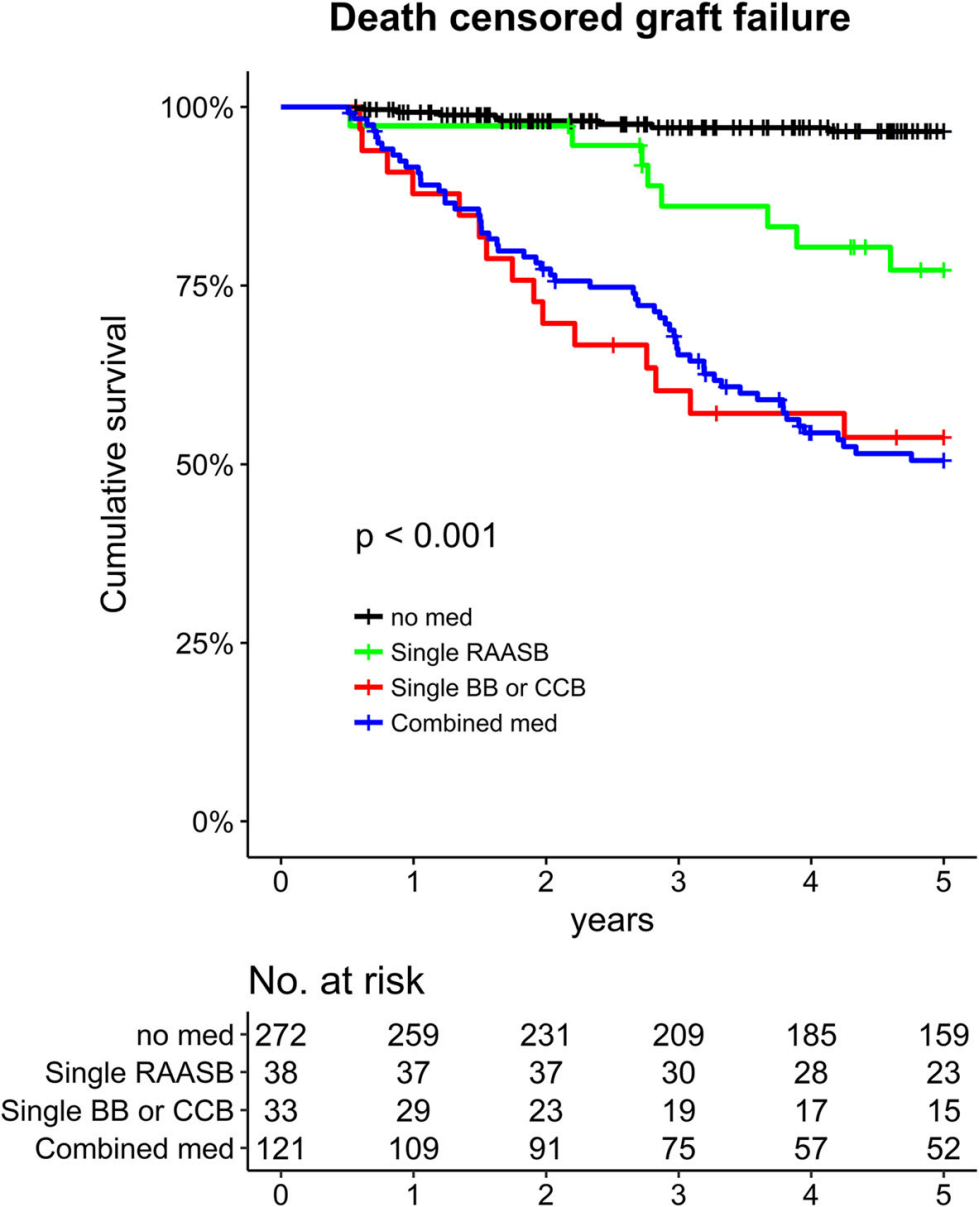
Kaplan-Meier survival curve showing death-censored graft failure according to the prescribed antihypertensive medication categories. The x-axis indicates time from the diagnosis of allograft IgAN while the y-axis indicates cumulative survival

Prognosis for DCGF was significantly different between the studied subgroups (Fig. 2). Patients who did not require antihypertensive medications had the best prognosis, as only 8/272 (2.9%) patients reached DCGF within 5 years of their allograft IgAN diagnosis. The single RAASB group demonstrated better outcome than the others, with 8/38 (21.1%) patients developing 5-year DCGF, compared to 15/33 (45.5%) and 52/121 (43.0%) in the single BB/CCB and combination groups, respectively.

The above results were repetitively observed in our regression analysis (Table 4). Compared to the single RAASB group, the no medication group demonstrated better prognosis, while the single BB/CCB and combination groups exhibited significantly worse prognoses. Single BB/CCB usage and its association with worse DCGF among single agent users remained valid when we included the time-averaged eGFR values. When we divided subgroups according to the presence of confirmed native IgAN, the inferiority for prognosis of single usage BB/CCB when compared with prognosis of single RAASB usage was shown only in allograft IgAN with unknown or clinical cause for ESRD (hazard ratio 3.16, 95% confidence interval 1.17–8.57, *P* = 0.02). On the contrary, in allograft IgAN cases with confirmed native IgAN, the usage of single BB/CCB showed non-significant difference regarding prognosis with the single RAASB usage (hazard ratio 2.40, 95% confidence interval 0.44–13.08, *P* = 0.31). In addition, within the combination group, the DCGF prognosis did not significantly differ according to RAASB usage (Additional file 3: Figure S3).

**Table 4:** Use of HTN medication within 6 months from allograft IgAN Dx and its’ association with the risk of DCGF

	Univariable		Multivariable – model 1^a^		Multivariable – model 2^b^	
	HR (95% CI)	*P*	Adjusted HR (95% CI)	*P*	Adjusted HR (95% CI)	*P*
Single RAASB	Ref.		Ref.		Ref.	
No medication	0.15 (0.06–0.40)	< 0.001	0.17 (0.06–0.48)	0.001	0.17 (0.06–0.49)	0.001
Single BB/CCB	2.74 (1.16–6.47)	0.02	3.05 (1.19–7.79)	0.02	2.88 (1.13–7.37)	0.03
Combination	2.76 (1.32–5.79)	0.007	2.72 (1.24–5.96)	0.01	2.28 (1.04–4.99)	0.04

*HR* Hazard ratio, *CI* Confidence interval, *RAASB* Renin-angiotensin-aldosterone system blockades, *BB* Beta blockers, *CCB* Calcium channel blockers

^a^Model 1. Adjusted for clinicopathologic characteristics at the time of allograft IgAN; age, sex, CKD-EPI eGFR (categorical, < 15, ≥15 and 30, ≥30 and < 45, ≥45 and < 60, ≥60), albuminuria (none or trace, 1+, and ≥ 2+), mean arterial pressure (mmHg), pathologic components of the Oxford classification (M, E, S, T, and C components), presence of acute rejection at the time of recurrence

^b^Model 2. The adjustment variable of eGFR value at allograft IgAN diagnosis in above Model 1 was substituted for time-averaged eGFR within 3 months after diagnosis of allograft IgAN

Finally, within the single RAASB group, DCGF was significantly better in those without high-degree albuminuria after 6 months from diagnosis (*P* = 0.048) (Additional file 4: Figure S4). Namely, allograft IgAN patients with high-degree of albuminuria after 6 months from their diagnosis demonstrated poor prognosis, with a 5-year DCGF rate of 5/13 (38.5%). By contrast, only 3/21 (14.3%) single RAASB users progressed to DCGF within 5 years in those without high-degree albuminuria after 6 months. Moreover, in our mediation analysis, high-degree albuminuria after 6 months was a partial mediator of the association between the antihypertensive medication subgroups and DCGF (*P* = 0.008).

## Discussion

In this retrospective study, we investigated the potential benefits of RAASB in allograft IgAN by identifying prescribed hypertensive medication categories and comparing the prognosis in terms of DCGF and changes in albuminuria levels. Our main finding was that allograft IgAN patients who received single RAASB demonstrated better graft outcome than those who received single BB/CCB or combination therapy. Among the single agent users, single RAASB usage was associated with a significantly reducing pattern of albuminuria. As the absence of high-degree dipstick albuminuria after 6 months was an important prognostic factor, benefits of RAASB on DCGF might have been related to its protective effect on urine albumin (a surrogate marker of urine protein) loss. However, the result was only significant in the allograft IgAN cases without confirmed native IgAN but was not evident in the recurred IgAN cases in that IgAN was also identified in native kidneys.

RAASB has been widely used for many renal diseases because of its protective effect on the cardiovascular and renal systems. The benefits of RAASB on renal prognosis were well-demonstrated within proteinuric primary glomerulonephritis or diabetic nephropathy patients [10, 11, 24, 25]. There is a possibility that the medication may also be beneficial for graft outcome after kidney transplantation; however, this has yet to be confirmed [13–16]. In addition, while clinical guidelines for kidney transplantation recipients suggest prescribing RAASB for those with recurrent glomerulonephritis and proteinuria [19], the recommendation level remains low. Our study showed possible benefits of RAASB usage as the initial single agent for better graft prognosis in allograft IgAN, which was not clearly indicated in a previous report [18]. In addition to the preceding study that showed RAASB’s protective effect on proteinuria among allograft IgAN patients [17], our study also demonstrated that the benefits may lead to better graft survival.

As stated, this potential benefit might be related to the identified medications’ protective effect on dipstick albuminuria, which is a surrogate marker of proteinuria [26, 27]. The reno-protective effect of RAASB is mainly derived from reduced intraglomerular pressure and proteinuria, which was considered to be independent of the underlying disease [17]. In the present study, high-degree albuminuria after certain period from the diagnosis of allograft IgAN was one of the most important predictors of DCGF, while MAP or baseline albuminuria demonstrated only weaker associations. This suggests that in a state of allograft IgAN, the severity of glomerulonephritis, which may be reflected by persistent albuminuria, may be an important parameter or damaging factor for worse graft prognosis. Considering the close linkage between single RAASB usage, decreased proportion of patients with high-degree albuminuria, and better DCGF, albuminuria reduction might be the moderator of the association between RAASB and better graft outcome among single agent users. In addition, this may also be supported by the fact failure to reduce albuminuria during the first 6 months from allograft IgAN diagnosis, even within the single RAASB group, was associated with worse allograft outcome. Clinicians should keep this result in mind and consider RAASB when single-agent antihypertensive therapy is required for allograft IgAN patients; however, they should also consider the side effects with caution [14, 28].

Still, it should be noted that the possible benefits of single agent RAASB over single BB or CCB was not confirmed herein for the recurred IgAN cases with confirmed native IgAN. In our previous study, the clinical significance of the Oxford classification was different according to allograft IgAN subtypes [20]. In allograft IgAN with confirmed native IgAN cases, M, E, S, and C components, which may reflect intraglomerular disease activity, were important prognostic parameters, but in the cases without confirmed native IgAN, only the T was a significant pathologic risk factor. When relating this with the current study results, appropriate immunosuppression may be more important in the “recurred IgAN” cases, which showed more rapid progression and in which pathologic parameters related to intraglomerular disease activity showed prognostic importance. On the other hand, in allograft IgAN cases without confirmed native IgAN, which were possibly de novo ones in which progressed tubular injury, the T, was an important prognostic factor, reduction of proteinuria to decrease progressive allograft injury might have been more important than in recurred IgAN cases. Our study suggests a future study investigating whether actual pathophysiology of recurred IgAN and de novo IgAN is different. Moreover, studying whether usage of immunosuppression is associated with difference in prognosis of recurred IgAN cases, particularly with high-risk pathologic parameters, may help developing individualized treatment strategy for allograft IgAN.

Patients who did not require antihypertensive medication following allograft IgAN diagnosis demonstrated better prognosis than those who did. By contrast, those who received combination therapy displayed poorer prognosis, regardless of the prescription of RAASB. A required medication dosage or the presence of uncontrolled BP might have been the more important parameter related to graft prognosis than the albuminuria in these patients. Furthermore, this implies that the benefit of RAASB usage in advanced allograft IgAN requiring multiple antihypertensive agents remains unclear. Therefore, the current study only supports the usage of RAASB when single-agent therapy is required.

Several unresolved limitations should be considered in our study. These are mainly related to the difficulty of conducting a study concerning post-transplant medication usage, especially for non-rejection allograft diseases. First, a hidden confounding effect might be present. It is true that clinicians may hesitate to prescribe RAASB in patients with rapidly declining graft function. Although we studied the long-term outcomes and attempted to adjust the potential confounders, this innate difference in antihypertensive medication indications might have affected our results. Second, as many patients were receiving combination therapy, a comparison between the single agents, which would give the most important consequence, was performed only in a limited number of patients. However, as this was one of the largest cohorts of allograft IgAN to date, this might be the only reported evidence that may demonstrate the possible benefits of RAASB usage on graft survival in allograft IgAN. Third, due to the study’s retrospective nature, whether the addition of RAASB in allograft IgAN patients is necessary could not be directly answered herein. In addition, several patients were already on RAASB, and a sufficient wash-out period could not have been secured. While a prospective study may be necessary to solve this problem, the inclusion of sufficient number of trial subjects may be an issue. Lastly, we used dipstick albuminuria values, a surrogate marker of proteinuria [21], and amounts of proteinuria were not measured using the 24-h collection or random urine protein/creatinine ratio. Although this is a significant limitation, the use of the variable was necessitated by the limited number of allograft IgAN patients with available quantified proteinuria results.

## Conclusions

The potential benefit of RAASB on allograft IgAN prognosis was highlighted in the present study. Clinicians may consider using RAASB for allograft IgAN cases without confirmed native IgAN, or de novo cases, particularly in those at an early stage who do not require combination therapy. Additional studies regarding the treatment strategies for primary glomerulonephritis in renal transplant recipients are warranted.

## Additional files

Additional file 1: **Figure S1.** Presence of high-degree albuminuria (≥ 2+) or high MAP (> 100 mmHg) and their association with 5-year DCGF within patients those did not receive any antihypertensive agents. (PDF 138 kb)

Additional file 2: **Figure S2.** Presence of high-degree albuminuria (≥ 2+) or high MAP (> 100 mmHg) and their association with 5-year DCGF within patients those did not receive any antihypertensive agents. (PDF 185 kb)

Additional file 3: **Figure S3.** 5-year DCGF according to usage of RAASB within the combination group. (PDF 106 kb)

Additional file 4: **Figure S4.** 5-year DCGF according to presence of a high-degree (≥ 2+) albuminuria at baseline or after 6 months from allograft IgAN diagnosis within the single RAASB group. (PDF 101 kb)

## Data Availability

The datasets used and/or analyzed during the current study are available from the corresponding author on reasonable request.

## Abbreviations

AMC: Asan medical center
BB: Beta blockers
BP: Blood pressure
CCB: Calcium channel blockers
DCGF: Death-censored graft failure
eGFR: Estimated glomerular filtration rate
ESRD: End stage renal disease
IgAN: Immunoglobulin A nephropathy
MAP: Mean arterial pressure
RAASB: Renin-angiotensin-aldosterone-system blockades
sCr: Serum creatinine
SNUH: Seoul national university hospital
