# Supplementary material for: Possible beneficial association between renin-angiotensin-aldosterone-system blockade usage and graft prognosis in allograft IgA nephropathy: a retrospective cohort study
Source: BMC Nephrol. 2019 Sep 11;20:354. doi: 10.1186/s12882-019-1537-1 (PMC6737644; doi:10.1186/s12882-019-1537-1)
Supplement: Supplementary file 1 — Figure S1. Presence of high-degree albuminuria (≥ 2+) or high MAP (> 100 mmHg) and their association with 5-year DCGF within patients those did not receive any antihypertensive agents. (PDF 138 kb) [file 12882_2019_1537_MOESM1_ESM.pdf]

Figure S1. Presence of high-degree albuminuria (≥ 2+) or high MAP (> 100 mmHg) and their association with 5-year DCGF within patients those did not receive any antihypertensive agents.

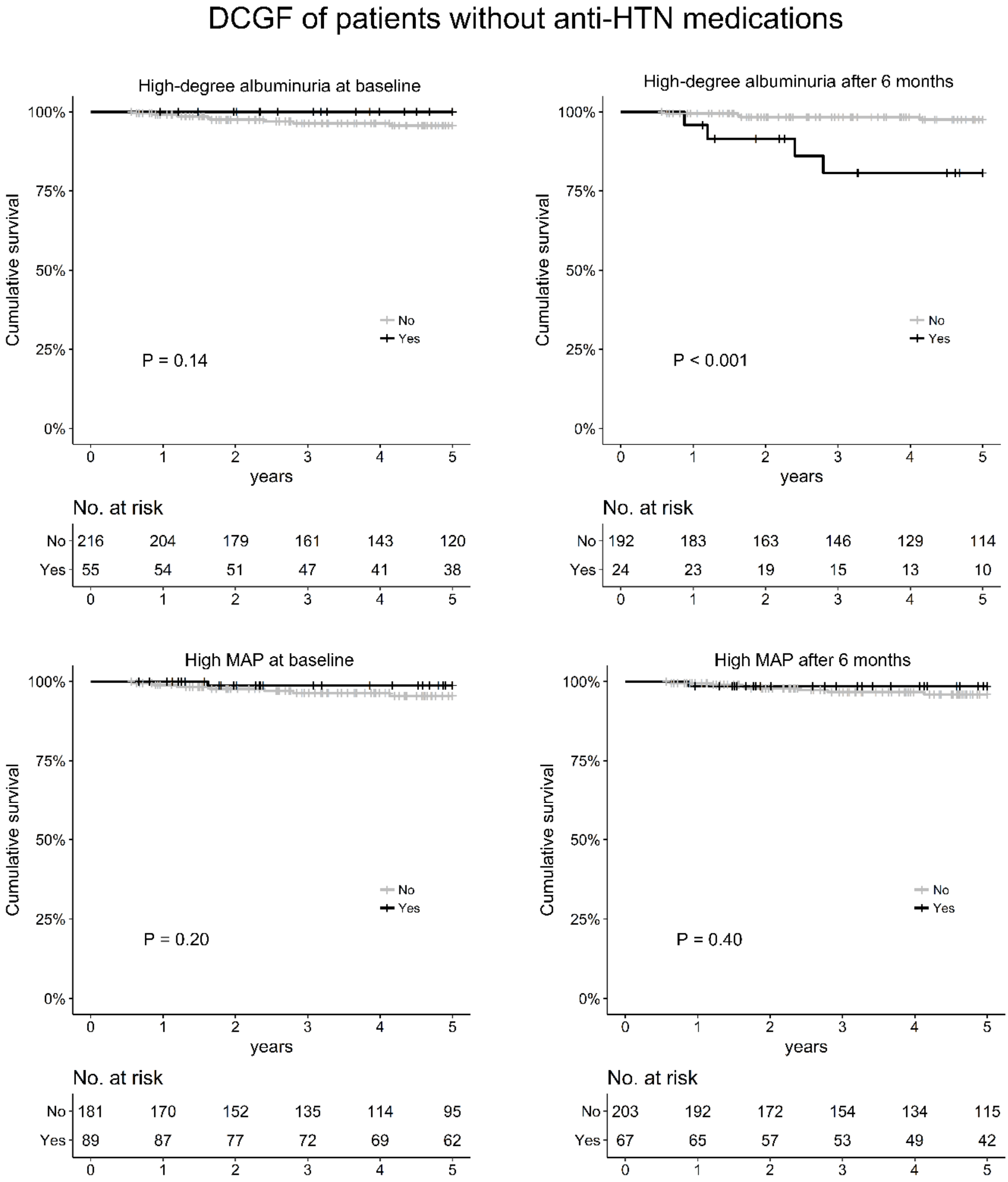

The variables at baseline and after 6 months from diagnosis are separately investigated. The x-axes indicate the years from allograft IgAN diagnosis and the y-axes indicate the cumulative survival.
