# Supplementary material for: Possible beneficial association between renin-angiotensin-aldosterone-system blockade usage and graft prognosis in allograft IgA nephropathy: a retrospective cohort study
Source: BMC Nephrol. 2019 Sep 11;20:354. doi: 10.1186/s12882-019-1537-1 (PMC6737644; doi:10.1186/s12882-019-1537-1)
Supplement: Supplementary file 3 — Figure S3. 5-year DCGF according to usage of RAASB within the combination group. (PDF 106 kb) [file 12882_2019_1537_MOESM3_ESM.pdf]

Figure S3. 5-year DCGF according to usage of RAASB within the combination group.

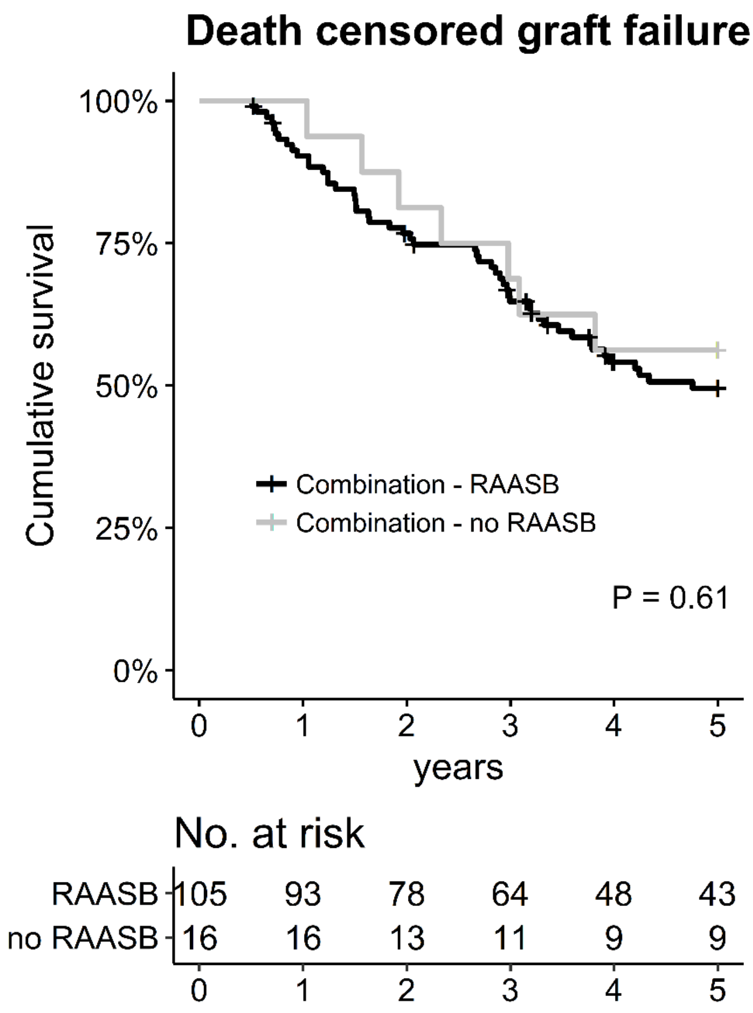

The x-axis indicates the time (years) from allograft IgAN diagnosis and the y-axis indicates the cumulative survival.
