# Supplementary material for: Possible beneficial association between renin-angiotensin-aldosterone-system blockade usage and graft prognosis in allograft IgA nephropathy: a retrospective cohort study
Source: BMC Nephrol. 2019 Sep 11;20:354. doi: 10.1186/s12882-019-1537-1 (PMC6737644; doi:10.1186/s12882-019-1537-1)
Supplement: Supplementary file 4 — Figure S4. 5-year DCGF according to presence of a high-degree (≥ 2+) albuminuria at baseline or after 6 months from allograft IgAN diagnosis within the single RAASB group. (PDF 101 kb) [file 12882_2019_1537_MOESM4_ESM.pdf]

Figure S4. 5-year DCGF according to presence of a high-degree (≥ 2+) albuminuria at baseline or after 6 months from allograft IgAN diagnosis within the single RAASB group.

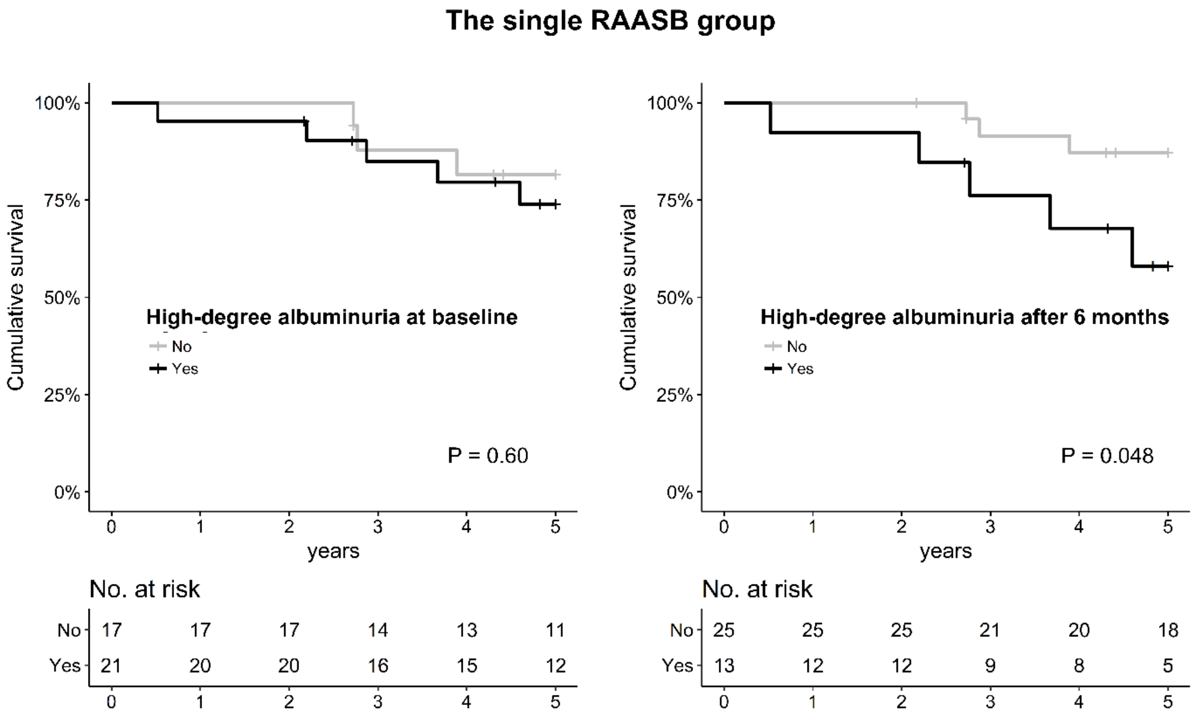

The x-axes indicated the time (years) from allograft IgAN diagnosis and the y-axes indicated the cumulative survival.
